# Development and Evaluation of the Adenoid/Nasopharynx Area Ratio on Lateral Cephalograms: Correlation With Nasal Endoscopy

**DOI:** 10.1002/oto2.70273

**Published:** 2026-06-29

**Authors:** Jiaming Zhu, Le Chen, Xiaoli Zhou, Wenyan Li

**Affiliations:** ^1^ Department of School of Health Science and Engineering University of Shanghai for Science and Technology Shanghai China; ^2^ Department of Otolaryngology–Head Neck Surgery Eye & ENT Hospital of Fudan University Shanghai China

**Keywords:** adenoid hypertrophy, adenoids/nasopharynx area ratio (ANAR), A/N ratio, lateral cephalometric radiography, nasal endoscopy, pediatric airway obstruction

## Abstract

**Objective:**

The clinical assessment of adenoid hypertrophy presents 2 practical challenges: (1) the need for an objective, reproducible estimate of nasopharyngeal obstruction in children when cooperation is limited and (2) the need for a reliable radiographic metric to support interdisciplinary assessment and communication between dental/orthodontic providers and otolaryngologists. To address these challenges, this study aimed to evaluate the diagnostic performance of a novel radiographic index—Adenoid/Nasopharynx Area Ratio—derived from lateral cephalometric radiographs.

**Study Design:**

Retrospective cohort study.

**Setting:**

Eye & ENT Hospital of Fudan University and affiliated orthodontic clinics, Shanghai, China (June 2023‐August 2024).

**Methods:**

Consecutive children aged 2 to 14 years flagged for suspected adenoid hypertrophy during routine dental examinations were screened; eligible participants were enrolled after prespecified exclusions. De‐identified data were analyzed between August and October 2024.

**Results:**

ANAR showed a numerically higher correlation with endoscopic grades than the A/N ratio (*r* = 0.649 vs 0.603). For severe obstruction, ANAR cutoff 0.64 yielded sensitivity 70.9% and specificity 82.3% (AUC 0.834; 95% CI, 0.76‐0.90); the A/N ratio AUC was 0.793. Inter‐rater reliability for ANAR measurement was good (intraclass correlation coefficient [A,1] = 0.924; 95% confidence interval, 0.848‐0.963; n = 30).

**Conclusion:**

Adenoid/Nasopharynx Area Ratio is a practical, reproducible planimetric index derived from lateral cephalograms that may support opportunistic upper airway assessment when imaging is already obtained for dental/orthodontic care. Nasal endoscopy remains the reference standard; ANAR is complementary, and additional radiographs solely for adenoid evaluation are not recommended.

Adenoid hypertrophy (AH) is a common issue during children's growth and development, representing a multidisciplinary condition, with a reported prevalence of approximately 34% in pediatric populations.^1^ Although its exact etiology remains unclear, AH is thought to arise from multifactorial interactions, including allergic inflammation, recurrent infections, and genetic predisposition.^2^, ^3^, ^4^, ^5^ Nasopharyngeal obstruction secondary to AH may result in chronic mouth breathing, snoring, and, in severe cases, obstructive sleep apnea (OSA), all of which adversely affect pediatric quality of life and long‐term health.^6^, ^7^, ^8^, ^9^, ^10^ Moreover, persistent nasopharyngeal obstruction due to AH may contribute to dentofacial developmental abnormalities.^11^, ^12^ Specifically, chronic mouth breathing can induce clockwise rotation of the mandible, promoting a Class II malocclusion and deep overbite over time.^13^ Affected children often present to the otolaryngology department with symptoms such as nasal congestion, mouth breathing, and snoring. Given these potential long‐term complications, early diagnosis and intervention are critical. Establishing a standardized screening system for AH could facilitate timely identification and individualized management, thereby mitigating the risk of maxillofacial developmental disorders and other secondary sequelae.^14^, ^15^, ^16^, ^17^, ^18^, ^19^ Although nasal endoscopy (NE) remains the gold standard for diagnosing AH,^20^, ^21^, ^22^, ^23^ its clinical application in pediatric populations has certain limitations. Because the examination is cooperation‐dependent and may be uncomfortable, tolerance can be limited in anxious or very young children due to fear or developmental factors.^24^ In addition to the presence of nasal secretions, breath‐holding behavior in children can alter soft palate position, potentially compromising the accuracy of adenoid size assessment during NE, as shown in Figure 1. As a noninvasive radiographic option, lateral cephalometric radiography continues to play a crucial role in AH evaluation.^23^, ^25^, ^26^, ^27^, ^28^, ^29^, ^30^, ^31^, ^32^ While the Adenoid/Nasopharynx (A/N) ratio is currently the primary radiographic metric, its correlation with endoscopic findings remains suboptimal.^33^, ^34^ To address this limitation, we propose a novel planimetric measurement approach: the Adenoid/Nasopharynx Area Ratio (ANAR).

**Figure 1:**
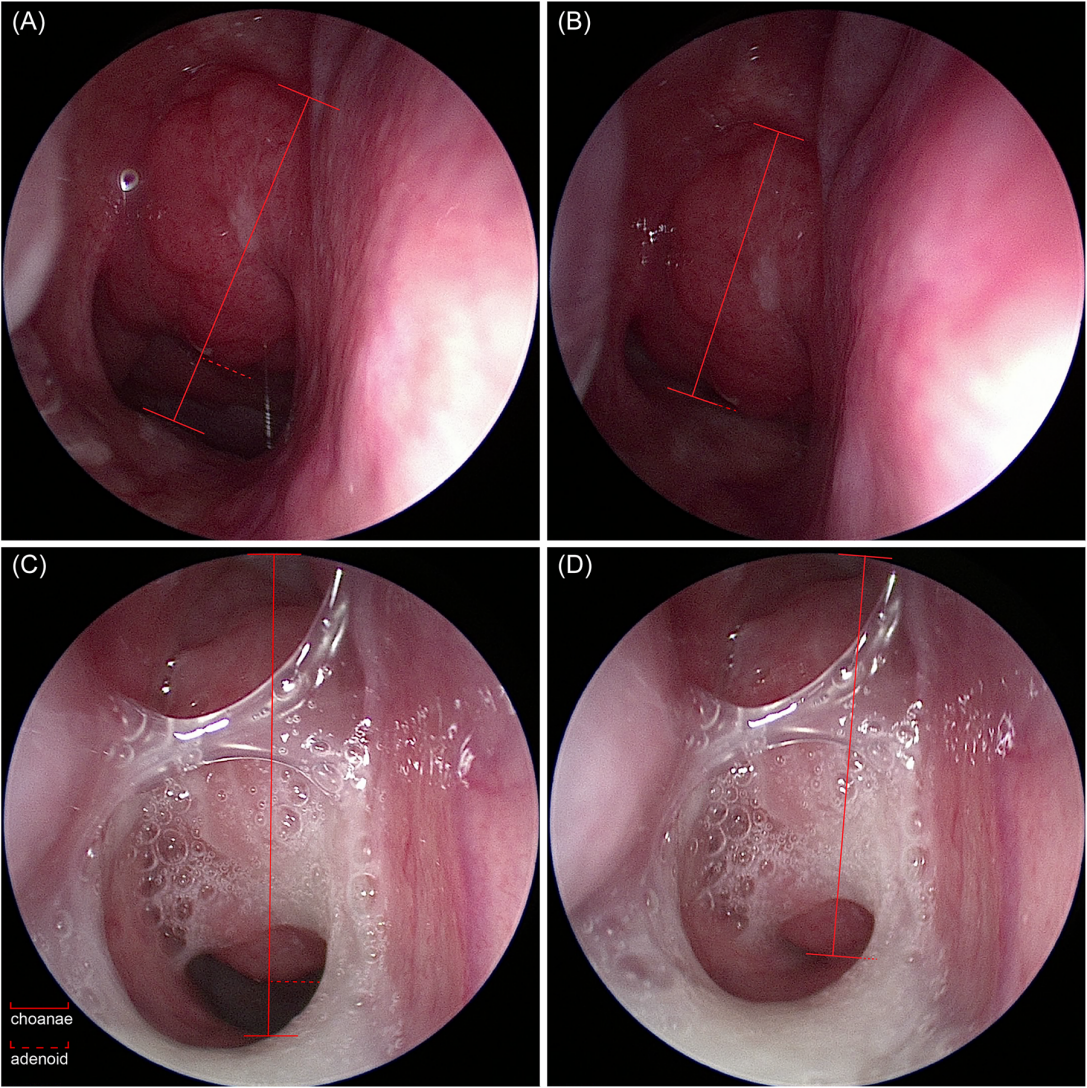
Influence of dynamic respiratory movements on the reliability of adenoid grading in nasal endoscopy. Endoscopic comparison of adenoid size under respiratory dynamics: (A, C) (inspiration) versus (B, D) (breath‐holding) in 2 representative patients.

This study aimed to preliminarily evaluate the diagnostic potential of ANAR in assessing AH severity and to examine its relationship with nasal endoscopic findings. We hypothesized that ANAR derived from routinely obtained lateral cephalograms would correlate with nasal endoscopic obstruction and provide a feasible adjunctive metric for assessing AH. Notably, our radiographic data were not obtained through additional imaging but were retrospectively collected from routine preorthodontic dental radiographs. These images, originally acquired for jaw development and occlusal relationship assessment, clearly depict nasopharyngeal anatomy. By correlating these radiographic findings with endoscopic results, our study demonstrates the feasibility of using routinely obtained cephalograms for opportunistic airway assessment and highlights a model for multidisciplinary collaboration in pediatric airway management.

## Material and Methods

### Participants

This retrospective study enrolled pediatric patients who underwent dental examinations at the Eye & ENT Hospital of Fudan University between June 2023 and August 2024. Through systematic chart review, we identified 117 AH cases meeting all of the following inclusion criteria:

1. Diagnosis of AH confirmed by NE, the gold standard, with the assessment performed by the same chief otolaryngologist.
2. Availability of original lateral nasopharyngeal radiographs.
3. Maximum one‐month interval between endoscopic and radiographic examinations.

This study was conducted in accordance with the Declaration of Helsinki and was approved by the Ethics Committee of Eye & ENT Hospital of Fudan University (No: 2024192).

### NE

NE was used as the reference standard for evaluating adenoid size and the degree of nasopharyngeal obstruction.^35^ Endoscopic assessment was performed by the same experienced chief otolaryngologist using a percentage‐based estimate of obstruction, recorded as the proportion of the nasopharyngeal airway occupied by adenoid tissue. For categorical analyses, obstruction was classified as Grade I (≤25%), Grade II (26%‐50%), Grade III (51%‐75%), and Grade IV (>75%). Severe hypertrophy was defined as Grade IV obstruction.^22^, ^36^

### Adenoids/Nasopharynx Ratio

The A/N ratio remains the most widely used radiographic parameter for evaluating adenoid size and nasopharyngeal obstruction severity.^37^, ^38^ The thickness of the adenoid (A) is defined as the vertical distance from the most prominent point of the adenoid to the lateral surface of the occipital bone slope. The width of the nasopharynx (N) is defined as the vertical distance from the perpendicular extension line of the adenoid's most prominent point to the posterior edge of the hard palate or the anterior mid‐upper margin of the soft palate.

According to the standards of the Journal of Clinical Radiology (China), an A/N ratio above 0.80 suggests significant hypertrophy.^39^

### ANAR

This study proposes the ANAR (Formula 1) as a new radiographic parameter for AH evaluation.

(1) $$\text{Adenoid}/\text{Nasopharynx Area Ratio}=\frac{\text{Anterior area of adenoid}}{\text{Anterior area of nasopharynx}}$$

**Figure 2:**
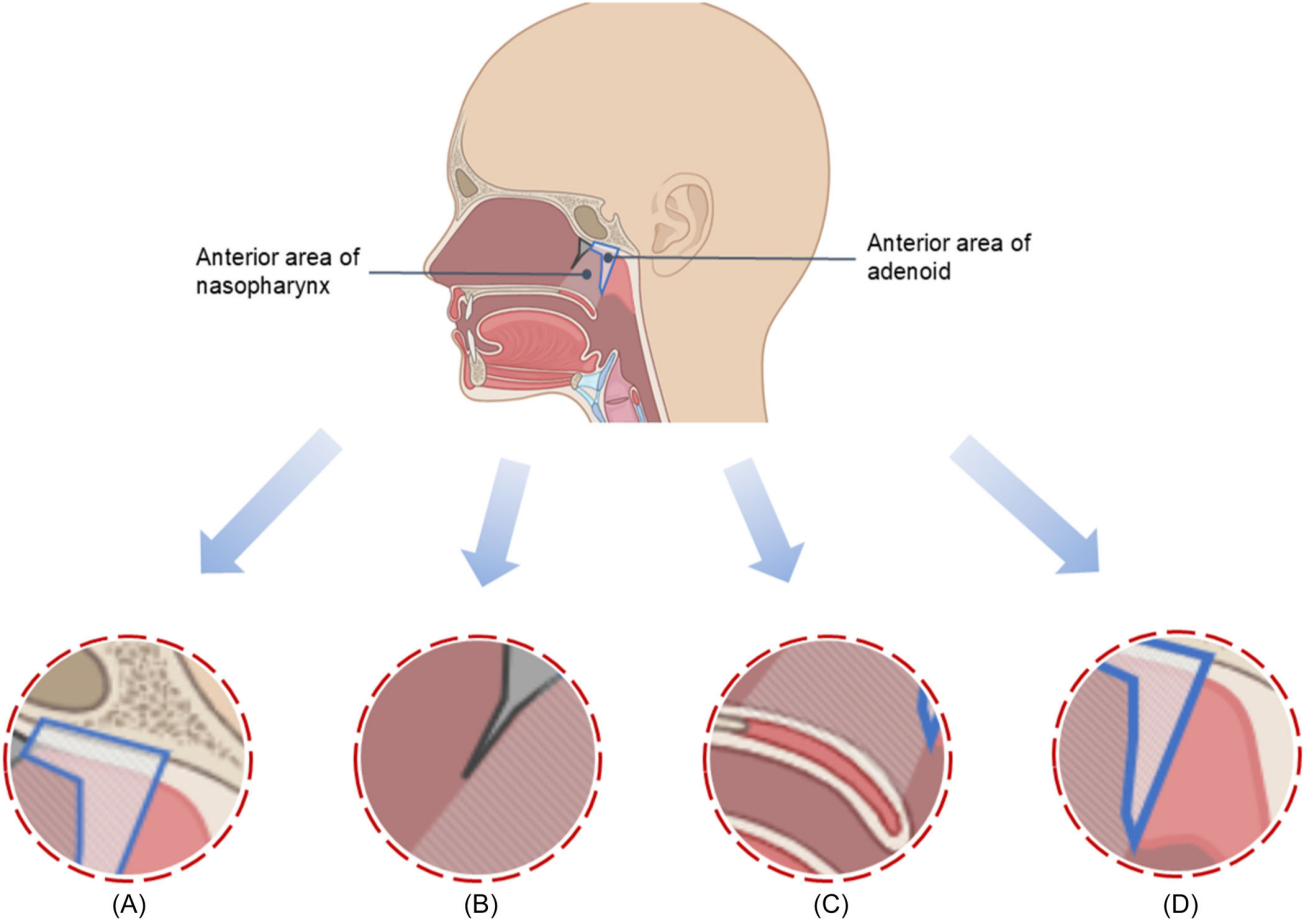
ANAR Measurement. (A) Tangent to the basioccipital surface. (B) Pterygoid plate tangent. (C) Basiocciput to hard–soft palate junction, then to line A. (D) Vertical from basiocciput to adenoid apex to the junction. ANAR, adenoid/nasopharynx area ratio.

**Figure 3:**
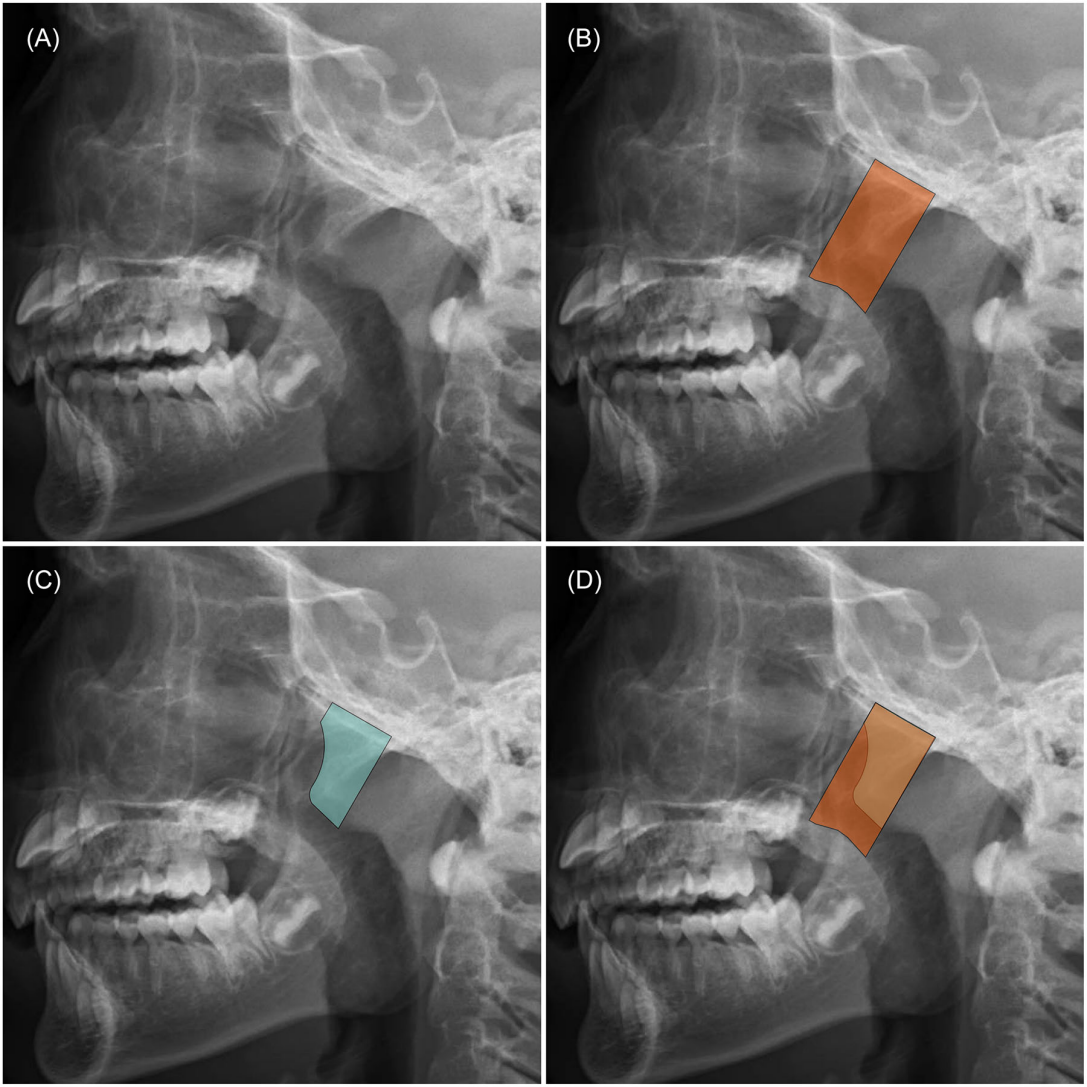
Methodology for calculating the ANAR in lateral cephalometric radiography. (A) original lateral nasopharyngeal radiographs. (B) Anterior area of nasopharynx. (C) Anterior area of adenoid. (D) ANAR. ANAR, adenoid/nasopharynx area ratio.

As shown in Figures 2 and 3, the anterior nasopharyngeal area is delineated by four linear boundaries:

1. A vertical line connecting the basioccipital surface to the adenoid's most prominent point, with its extension to the hard/soft palate junction.
2. The tangent line along the basioccipital surface.
3. The tangent line of the pterygoid plate.
4. A connecting line extends from the basioccipital surface to the hard/soft palate junction, then joins line 1. A step‐by‐step illustration of boundary definition and ImageJ‐based tracing is provided in Supplemental Figure S1, available online.

Radiographic measurements (A/N ratio and ANAR) were performed on de‐identified cephalograms by an investigator blinded to NE findings (endoscopy grades were inaccessible until measurements were completed); within the predefined region, adenoid area was obtained in ImageJ by manual ROI tracing of the irregular contour, with automatic computation of the enclosed area.

### Statistical Analysis

Statistical analysis was carried out using SPSS 20.0 software and R (version 4.5.2). Spearman's rank correlation coefficients were calculated to assess the associations between NE obstruction grade and (1) the A/N ratio and (2) ANAR. The difference between these two dependent (overlapping) correlations sharing one variable (endoscopy) was evaluated using Williams' test (two‐sided).

Using NE as the reference standard, patients were dichotomized as positive or negative based on endoscopic obstruction >75% (Grade IV hypertrophy). Receiver operating characteristic (ROC) curves were constructed for the A/N ratio and ANAR, and the area under the ROC curve (AUC) with corresponding confidence intervals was calculated. The optimal cut‐off value was determined using the Youden index, and sensitivity and specificity were reported. AUCs were compared using DeLong's test for two correlated ROC curves (paired design).

Inter‐rater reproducibility of ANAR measurements was assessed in a subset of 30 cases independently measured by 2 raters, using the intraclass correlation coefficient (ICC) with a two‐way model, absolute agreement, and single measurements (ICC[A,1]). A two‐sided *P* < .05 was considered statistically significant.

## Results

### Study Population

The study cohort comprised 117 consecutive pediatric patients (male‐to‐female ratio = 1:1.02) aged 2 to 14 years, with a median age of 6 years (mean ± SD: 6.44 ± 2.87 years).

**Figure 4:**
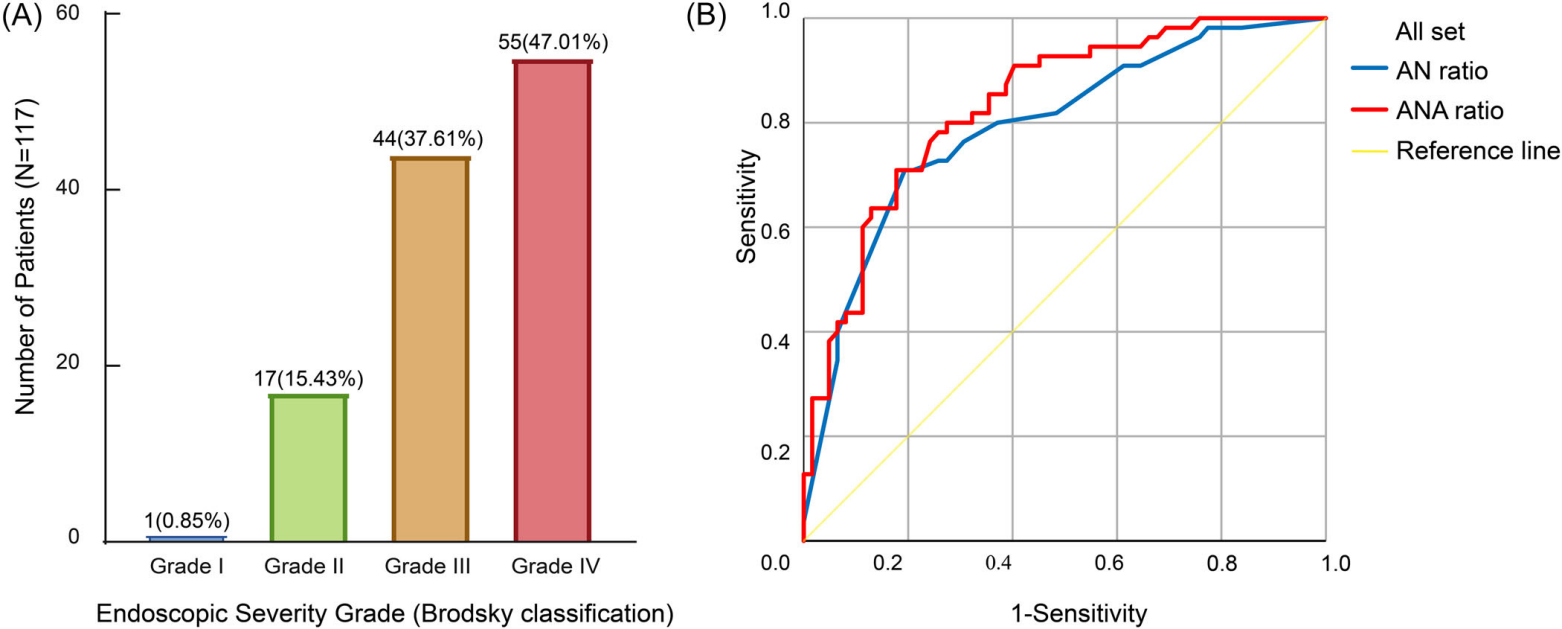
(A) Distribution of endoscopic severity grades in pediatric patients with adenoid hypertrophy. (B) ROC curve. AN, Adenoids/Nasopharynx; ANAR, Adenoids/Nasopharynx area ratio; ROC, receiver operating characteristic.

Based on nasal endoscopic obstruction grades, patients were stratified into 4 severity categories:

1. Grade I (≤25% obstruction): 1 patient (0.85%).
2. Grade II (26%‐50% obstruction): 17 patients (14.53%).
3. Grade III (51%‐75% obstruction): 44 patients (37.61%).
4. Grade IV (>75% obstruction): 55 patients (47.01%).

as shown in Figure 4A.

### Measurement reliability

Inter‐rater reliability for ANAR was good, with ICC = 0.924 (95% CI, 0.848‐0.963) based on 30 repeated measurements.

### Spearman Correlation Coefficient with NE

This study employed 2 radiographic assessment methods (ANAR and A/N ratio) to evaluate 117 patients, with correlation analyses performed against the nasal endoscopic findings. Given the nonnormal distribution of quantitative variables, Spearman's rank correlation analysis was implemented.

Both indices showed statistically significant, moderate positive correlations with endoscopic grading (*r* = 0.649 for ANAR [95% CI, 0.52‐0.75] and 0.603 for the A/N ratio [95% CI, 0.47‐0.71]; both *P* < .05). Although ANAR was numerically higher, the difference between these dependent correlations was not statistically significant (Williams' test: Δ*ρ* = 0.046; *P* = .413).

### ROC Curve

In this study, patients with endoscopically confirmed grade IV AH (n = 55, 47.01%) were classified as the positive group, while those with grade I to III hypertrophy (n = 62, 52.99%) comprised the negative group.

In the diagnostic performance of the A/N ratio, the optimal cutoff value is 0.74 when the Youden index is 0.515, with a sensitivity of 0.709 and a specificity of 0.806. The AUC for the corresponding ROC is 0.793.

And in the diagnostic performance of the ANAR, the optimal cutoff value is 0.64 when the Youden index is 0.532, with a sensitivity of 0.709 and a specificity of 0.823. The AUC for the corresponding ROC is 0.834. Results are summarized in Table 1 and Figure 4B.

**Table 1:** The AUC, YI, Cut‐off Point, Se and Sp of A/N ratio and ANAR Examinations for the Entire Cohort of Patients

	AUC (95% CI)	Yi (95% CI)	Cut‐off Point (95% CI)	Se (95% CI)	Sp (95% CI)
A/N ratio	0.793	0.515	0.74	0.709	0.806
ANAR	0.834	0.532	0.64	0.709	0.823

Abbreviations: A/N, Adenoid/Nasopharynx; ANAR, Adenoids/Nasopharynx area ratio; CI, confidence interval.

AUCs were compared using DeLong's test for paired ROC curves, which showed no statistically significant difference (ΔAUC = 0.041; 95% CI, −0.033 to 0.116; *P* = .278).

## Discussion

This preliminary study found that ANAR showed a numerically higher correlation with nasal endoscopic obstruction grades (*r* = 0.649) than the conventional A/N ratio (*r* = 0.603); however, the difference between these dependent correlations was not statistically significant (Williams' test, *P* = .413). These results suggest that ANAR provides a feasible, anatomically grounded planimetric measure of adenoid‐related nasopharyngeal obstruction on lateral cephalograms.

When adenoid tissue occupies more than 75% of the nasopharynx on NE, severe obstruction is generally considered to be present.^22^ Therefore, this study used an endoscopic obstruction threshold of 75% to classify patients as negative (≤75%) or positive (>75%) in the ROC analyses. As summarized in Table 1 and Figure 4, ANAR and the A/N ratio yielded the same sensitivity at their respective optimal cutoffs, while ANAR showed slightly higher specificity; the difference in AUCs was not statistically significant by DeLong's test (*P* = .278).

The conventional A/N ratio method demonstrates significant correlation with nasal endoscopic findings (*r* = 0.603), consistent with prior studies by Mlynarek et al and Nawal's group.^35^, ^40^ In our cohort, ANAR demonstrated a numerically higher correlation (*r* = 0.649), although the difference between correlations was not statistically significant. This discrepancy likely stems from the A/N ratio's fundamental limitation of measuring only the narrowest airway dimension (linear measurement), whereas the ANAR method (Figure 2) provides a more comprehensive evaluation through two‐dimensional area assessment of the entire nasopharyngeal obstruction. In our experience, ANAR generally requires approximately 3 to 4 minutes per case, whereas the A/N ratio can usually be obtained within approximately 2 minutes, depending on boundary clarity and operator familiarity. Because ANAR is based on planimetric area delineation, it naturally aligns with image segmentation workflows and may facilitate future development of automated or artificial intelligence–assisted quantification of AH.

Previous work by Soldatova et al^30^ established the clinical utility of lateral cephalometric radiography (using the A/N ratio method) for quantifying AH in symptomatic patients. Our study extends these findings by evaluating a planimetric area‐based index and quantifying its reproducibility. In ROC analyses for severe obstruction, ANAR showed a numerically higher AUC than the A/N ratio (0.834 vs 0.793), although the difference was not statistically significant (DeLong's test, *P* = .278). Therefore, ANAR should not be interpreted as clearly superior to the conventional A/N ratio. Rather, its potential value may lie in standardized area‐based quantification and opportunistic use when cephalograms are already available.

When symptoms alone are inconclusive and tolerance of NE is limited, ANAR derived from already available lateral cephalograms may provide complementary quantitative information for upper‐airway assessment. Nevertheless, NE remains the reference standard for definitive evaluation.

The application of cone beam computed tomography (CBCT) in adenoid assessment enables accurate visualization of true airway dimensions through 3D reconstructions and a volume‐rendering technique,^41^, ^42^, ^43^ demonstrating high concordance with nasopharyngeal endoscopic measurements.^44^ However, the radiation exposure associated with cone CBCT cannot be overlooked in pediatric examinations, as excessive exposure may increase the risk of hematologic malignancies and solid tumors.^45^, ^46^, ^47^, ^48^, ^49^ Therefore, CBCT is not recommended as a routine diagnostic method for AH.

Limitations of this study include that it was not specifically powered to detect small differences between radiographic indices; therefore, a modest true difference between ANAR and the A/N ratio cannot be excluded. Standardized symptom severity data (eg, validated questionnaires or structured symptom scores) were not consistently available in this retrospective cohort, precluding a robust analysis of symptom–endoscopy or symptom–radiograph correlations. Similar to the A/N ratio method for assessing adenoid size, the proposed ANAR method requires a considerable amount of time for labeling on lateral cephalometric radiography. Additionally, it cannot avoid the disadvantages associated with lateral cephalometric radiography, such as radiation exposure, potential overlap of anatomical structures, and respiratory effects.^50^ Although inter‐rater reliability for ANAR was good, further validation in larger multi‐reader and multicenter datasets is warranted.

## Conclusion

ANAR provides a practical, anatomically grounded, and reproducible planimetric quantification of adenoid‐related nasopharyngeal obstruction on lateral cephalograms. In this cohort, ANAR showed a moderate association with nasal endoscopic obstruction grades and comparable discrimination to the conventional A/N ratio for severe obstruction. ANAR may enhance the clinical utility of cephalometric radiographs for opportunistic upper airway assessment when imaging is already obtained for dental/orthodontic care. NE remains the reference standard, and ANAR should be considered complementary; additional radiographs solely for adenoid evaluation are not recommended.

## Author Contributions

**Jiaming Zhu,** contributed to design, data analysis and interpretation, drafted the manuscript; **Le Chen,** contributed to conception and design, data acquisition and interpretation, drafted the manuscript; **Xiaoli Zhou,** contributed to data acquisition and drafted manuscript; **Wenyan Li,** contributed to conception and critically revised manuscript; **Jiaming Zhu** and **Le Chen,** contributed equally to this work and should be considered co‐first authors; all authors gave their final approval and agree to be accountable for all aspects of the work.

## Disclosures

### Competing interests

None.

### Funding source

None.

## Supporting information


**Supplementary Fig. S1.** Stepwise ImageJ workflow for ANAR on lateral cephalograms: opening the image, drawing reference lines, delineating the anterior nasopharyngeal airway and adenoid areas, measuring both areas, and calculating ANAR.
