# Supplementary figures and images for: Development and Evaluation of the Adenoid/Nasopharynx Area Ratio on Lateral Cephalograms: Correlation With Nasal Endoscopy
Source: OTO Open. 2026 Jun 29;10(3):e70273. doi: 10.1002/oto2.70273 (PMC13312990; doi:10.1002/oto2.70273)

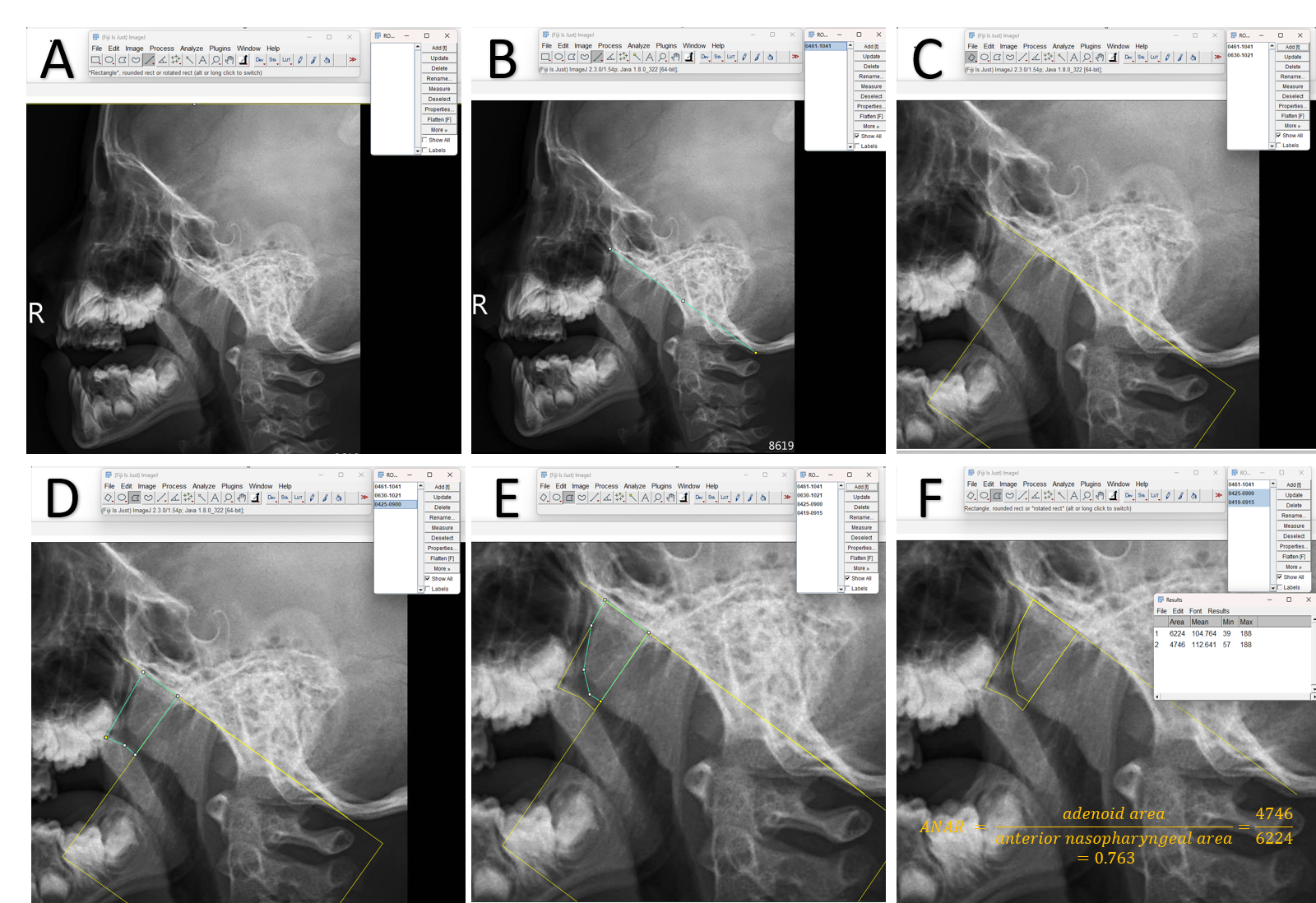

Supplement: Supplementary file 1 — Supplementary Fig. S1. Stepwise ImageJ workflow for ANAR on lateral cephalograms: opening the image, drawing reference lines, delineating the anterior nasopharyngeal airway and adenoid areas, measuring both areas, and calculating ANAR. [file OTO2-10-e70273-s001.tif]
